# Supporting the implementation of stroke quality-based procedures (QBPs): a mixed methods evaluation to identify knowledge translation activities, knowledge translation interventions, and determinants of implementation across Ontario

**DOI:** 10.1186/s12913-018-3220-9

**Published:** 2018-06-18

**Authors:** Julia E. Moore, Christine Marquez, Kristen Dufresne, Charmalee Harris, Jamie Park, Radha Sayal, Monika Kastner, Linda Kelloway, Sarah E. P. Munce, Mark Bayley, Matthew Meyer, Sharon E. Straus

**Affiliations:** 1 Li Ka Shing Knowledge Institute, St. Michael’s Hospital, 30 Bond Street, Toronto, ON M5B 1W8 Canada; 2 North York General Hospital, 4001 Leslie St, Toronto, ON M2K 1E1 Canada; 3 Cardiac Care Network of Ontario, 4100 Yonge Street, Toronto, ON M2P 2B5 Canada; 4 Toronto Rehabilitation Institute, University Health Network, 550 University Avenue, Toronto, ON M5G 2A2 Canada; 5 Cardiac Care Network, Department of Epidemiology and Biostatistics, Western University, 1151 Richmond St, Ontario, London N6A 5C1 Canada; 6 Department of Medicine, Faculty of Medicine, University of Toronto, 1 King’s College Circle, Medical Sciences Building, Toronto, ON M5S 1A8 Canada; 7 The Center for Implementation, 20 Northampton Dr, Toronto, ON M9B 4S6 Canada

**Keywords:** Knowledge translation activities, Implementation, Stroke guidelines, Knowledge to action, KT interventions, Quality-based procedures, Mixed-methods evaluation

## Abstract

**Background:**

In 2013, Health Quality Ontario introduced stroke quality-based procedures (QBPs) to promote use of evidence-based practices for patients with stroke in Ontario hospitals. The study purpose was to: (a) describe the knowledge translation (KT) interventions used to support stroke QBP implementation, (b) assess differences in the planned and reported KT interventions by region, and (c) explore determinants perceived to have affected outcomes.

**Methods:**

A mixed methods approach was used to evaluate: activities, KT interventions, and determinants of stroke QBP implementation. In Phase 1, a document review of regional stroke network work plans was conducted to capture the types of KT activities planned at a *regional* level; these were mapped to the knowledge to action framework. In Phase 2, we surveyed Ontario hospital staff to identify the KT interventions used to support QBP implementation at a*n organizational* level. Phase 3 involved qualitative interviews with staff to elucidate deeper understanding of survey findings.

**Results:**

Of the 446 activities identified in the document review, the most common were ‘dissemination’ (24.2%; *n* = 108), ‘implementation’ (22.6%; *n* = 101), ‘implementation planning’ (15.0%; *n* = 67), and ‘knowledge tools’ (10.5%; *n* = 47). Based on survey data (*n* = 489), commonly reported KT interventions included: staff educational meetings (43.1%; *n* = 154), champions (41.5%; *n* = 148), and staff educational materials (40.6%; *n* = 145). Survey participants perceived stroke QBP implementation to be successful (median = 5/7; interquartile range = 4–6; range = 1–7; *n* = 335). Forty-four people (e.g., managers, senior leaders, regional stroke network representatives, and frontline staff) participated in interviews/focus groups. Perceived facilitators to QBP implementation included networks and collaborations with external organizations, leadership engagement, and hospital prioritization of stroke QBP. Perceived barriers included lack of funding, size of the hospital (i.e., too small), lack of resources (i.e., staff and time), and simultaneous implementation of other QBPs.

**Conclusions:**

Information on the types of activities and KT interventions used to support stroke QBP implementation and the key determinants influencing uptake of stroke QBPs can be used to inform future activities including the development and evaluation of interventions to address barriers and leverage facilitators.

**Electronic supplementary material:**

The online version of this article (10.1186/s12913-018-3220-9) contains supplementary material, which is available to authorized users.

## Background

In 2012, Ontario’s Action Plan for Health Care was launched to transform how health care was delivered by addressing the province’s demographic and fiscal challenges [1]. To support this plan, the Health System Funding Reform (HSFR) was introduced to change how hospitals were funded, with the goal of aligning health spending with quality and sustainability. A major component of the HSFR was the development of evidence-informed quality-based procedures (QBPs), which were identified using expert panels convened by Health Quality Ontario (HQO), and the ADAPTE guidelines adaptation approach [2, 3]. The goal of QBPs is to promote the use of evidence-based practices within targeted clinical groups that require similar care, thereby standardizing care, improving quality, and reducing system inefficiencies. By the end of 2015, HQO had developed 20 QBPs, including a clinical handbook for each.

The current study focuses on the implementation of stroke QBPs, which are evidence-based guidelines for stroke. Stroke care costs the Ontario economy over $1.1 billion annually [4]. Stroke QBPs include best practices in early assessment procedures (e.g., using a standardized stroke scale), acute care (e.g., determining eligibility for treatment with intravenous tissue plasminogen activator), and rehabilitation (e.g., receiving at least 3 h of direct task-specific therapy per day). It was anticipated that standardization of these procedures (i.e., implementation of QBPs) would improve the quality of stroke care that an estimated 25,500 patients receive each year [4].

To effectively implement QBPs (i.e., evidence-based stroke guidelines) and produce desired best practices, changes at multiple levels in the health care system are required including, clinician practice changes, organizational changes, and system/structural changes. In 2013, HQO provided hospitals with the Clinical Handbook for Stroke to support stroke guideline implementation [5]. The handbook includes recommended groupings of patients defined by stroke severity, and clinical best practice recommendations. The implementation of stroke QBPs was unique compared with other QBPs because it is supported by the Ontario Stroke Network (OSN). The province of Ontario consists of 14 Local Health Integration Networks (LHINs), which provide planning and support to regional health care organizations. The province’s stroke system has been sub-divided into 11 Regional Stroke Networks (RSNs). The RSNs provides leadership, development, implementation, and integration of stroke care across the regions and along the continuum of stroke care. The OSN supports the RSNs and LHINs in measuring and reporting on performance, driving continuous improvement, partnering to achieve best practices, and creating innovations for stroke prevention, care, recovery, and reintegration. The OSN also serves as a hub for knowledge exchange and best practice implementation. Unfortunately, even when evidence-based guidelines and knowledge tools are available, this does not necessarily result in a change in practice or outcomes. Knowledge translation (KT) provides guidance on how to support the implementation of guidelines in practice [6]. KT involves doing a series of process steps and using interventions to change behavior [7]. Specifically, process models describe the iterative steps (i.e., KT activities) to use research in practice (see Table 1); examples of **KT activities** include conducting a needs assessment, assessing barriers and facilitators to evidence implementation, disseminating the handbook, and developing partnerships. **KT interventions** are the interventions (e.g., education, reminders, audit and feedback) used to change behaviour to align with the best practices (e.g., the stroke QBP). Selecting and delivering KT interventions are examples of KT activities. Research has been conducted on the barriers and facilitators to implementing stroke guidelines [8–10] and as well as the evaluation of the implementation of stroke guidelines [11], but to our knowledge, no research has used KT models and frameworks.

**Table 1:** Definitions of KT activities [6] and KT interventions [8]

	Definitions
KT Activities [6]	KT activities are those used in the process of using research evidence (stroke recommendations) in practice such as conducting a needs assessment, assessing barriers and facilitators, disseminating the handbook and, developing partnerships.
Knowledge tools	Refining knowledge for decision-making (e.g., clinical practice guidelines, decision aids, algorithms)
Identify problem/ identify, review, select knowledge	Identification of the knowledge-to-action gaps (knowledge needs) as a starting point of knowledge implementation. Involves rigorous methods and engagement with relevant stakeholders
Adapt knowledge to local context	Adapting the knowledge to the local settings to make sure it is relevant and feasible (e.g., customizing clinical practice guideline for a particular organization)
Assess barriers/facilitators to knowledge use	Assessing areas that impede and facilitate the uptake of knowledge
Select, tailor, implement interventions - implementation planning	Plans to select and tailor interventions to the identified barriers and facilitators
Select, tailor, implement interventions - implementation	Selecting and tailoring interventions to the identified barriers and facilitators
Monitor knowledge use	Defining what constitutes knowledge use so it can be measured (i.e., conceptual, instrumental, strategic) Determining the extent to which the interventions have been successful in bringing about change
Evaluate outcomes	Determining the impact of using the knowledge using explicit, rigorous methods
Sustain knowledge use	Continued implementation of evidence over time, can include assessing barriers to knowledge sustainability; tailoring interventions to these barriers; monitoring ongoing knowledge use; evaluating initial and sustained use
Other – dissemination	The purposeful spreading or distribution of knowledge or research to a specific audience, such as is done in scientific journals and at scientific conferences.
Other – stakeholder engagement	Actively engaging key stakeholders throughout the implementation process, and forming and sustaining positive and productive collaborations.
KT interventions [8]	KT interventions are the interventions (e.g. education, reminders, audit and feedback) used to change behaviour to align with the best practices (e.g., the QBP).
Accreditation	Process of review to demonstrate the ability to meet predetermined criteria and standards of accreditation established by a professional accrediting agency (e.g., Stroke Distinction)
Changes in physical structure, facilities, and equipment	A change of location of clinical work stations, inclusion of equipment where technology in question is used in a wide range of problems and is not disease specific
Changes in quality monitoring system	Presence and organization of quality monitoring mechanisms
Changes in setting/site of delivery	A change in care delivery location (e.g., moving a family planning service from a hospital to a school)
Skill mix changes	Changes in numbers, types or qualifications of staff
Audit and feedback	Any summary of clinical performance of health care over a specified period of time. The summary may also have included recommendations for clinical action. The information may have been obtained from medical records, computerized databases, or observations from patients
Champion/opinion leader	Use of providers nominated by their colleagues as being influential in changing behaviour
Continuity of stroke care	An intervention which includes one or many episodes of care for inpatients or outpatients. Continuity of care also includes arrangements for follow-up and case management, including co-ordination of assessment, treatment and arrangement for referrals
Educational meetings	Participation in conferences, lectures, workshops or traineeships
Local consensus processes	Inclusion of participating providers in discussion to ensure that they agreed that the chosen clinical problem was important and the approach to managing the problem was appropriate
Multidisciplinary teams	Creation of a new team of health professionals of different disciplines or additions of new members to the team who work together to care for patients
Patient educational materials	Distribution of published or printed recommendations for patients, including clinical practice guidelines, audio-visual materials and electronic publications.
Reminders	Patient or encounter specific information, provided verbally, on paper or on a computer screen, which is designed or intended to prompt a health professional to recall information. This would usually be encountered through their general education; in the medical records or through interactions with peers, and so remind them to perform or avoid some action to aid individual patient care. Computer aided DS is included
Revision of professional roles	Also known as ‘professional substitution’, specialist role’ or ‘boundary encroachment’, this includes the shifting of roles among health professionals and expansion of role to include new tasks. See also revision of professional roles – nursing and revision of professional roles – pharmacy intervention categories for specified nursing or pharmacy led care
Staff educational materials	Distribution of published or printed recommendations for staff, including clinical practice guidelines, audio-visual materials and electronic publications.

The purpose of this study was to understand the impact of stroke QBP implementation in hospitals across Ontario, including factors that may have affected successful implementation. In particular, we aimed to describe the KT activities and KT interventions used to support stroke QBP implementation, to assess differences in the planned and reported KT interventions by region, and to explore the determinants perceived to have affected the success of implementation.

## Methods

### Design/approach

We used a three-phase mixed methods evaluation approach. Four frameworks were used to understand and categorize KT activities, KT interventions, and the factors affecting implementation [7]. The knowledge to action (KTA) framework was used to guide our understanding of the types of KT activities and KT interventions that were planned. The KTA is a process model based on a systematic review of over 30 planned action theories that outlines the process involved in implementing evidence in practice [6]. The Cochrane Effective Practice and Organization of Care (EPOC) Group’s taxonomy of KT interventions framework was used to evaluate QBP implementation activities [12]. This taxonomy was used to categorize KT interventions; there are accompanying systematic reviews for the categories describing their effectiveness at changing practice [13]. To guide our analysis and understanding of the determinants perceived to have influenced the success of stroke QBP implementation, we used the consolidated framework for implementation research (CFIR) and the theoretical domains framework (TDF). The TDF addresses *individual* level barriers and facilitators shown to influence behaviour; the CFIR inner and outer setting domains describe *organizational* level barriers [14, 15].

In Phase 1, a review of RSN work plan documents was conducted to capture the types of KT activities and interventions planned at a *regional* level. In Phase 2, survey data from hospital staff across the province were used to describe the KT interventions and resources used to support QBP implementation at a*n individual and organizational* level. Survey data from Phase 2 were also used to gain a broad understanding of stakeholders’ perception of the success of stroke QBP implementation and the factors that may have affected implementation outcomes at *an individual and organizational* level. Phase 3 involved qualitative interviews and focus group sessions with staff to help elucidate deeper descriptions of survey findings. Data triangulation involved comparing the KT interventions described at an *organizational* level to those planned at a *regional* level (Phases 1 and 2), with Phase 3 interviews used to explore key findings.

### Phase 1: Document review

#### Document review data abstraction

A document review was conducted on the regional work plans (i.e., work plans that summarize the actions to be taken to implement QBPs) from each of the 11 RSNs. The data abstraction template was piloted by three reviewers who independently used the template to review work plans from one region selected at random; the reviewers then deliberated and refined the template based on usability of the template and emerging data. Data were then abstracted in duplicate by two members of the study team on the following categories: strategic priorities, objectives and goals, deliverables (planned or completed), deliverable target dates, notes on completion of deliverables, specific implementation site, and department. If the information was not available that element was left blank.

Each deliverable from the work plan was mapped to one of 12 KT activities (see Table 1 for definitions). The KTA model was the basis for this mapping as it presents iterative stages used to move evidence into practice (knowledge synthesis; knowledge tools; identify problem/identify, review, select knowledge; adapt knowledge to local context; assess barriers/facilitators to knowledge use; select, tailor, implement; monitor knowledge use; evaluate outcomes; and sustain knowledge use). Two KT activities not explicitly presented as KTA stages were added: dissemination and stakeholder engagement. Using an integrated KT approach, stakeholders are engaged throughout the process [16]; dissemination activities may be included in the “implement” stage or following the development of knowledge tools. Additionally, the “select, tailor, implement interventions” stage was split into two activities: implementation planning and implementation execution to differentiate between future and current activities. A second mapping activity was conducted on the “select, tailor, implement interventions- implementation” stage of the KTA. These deliverables were further sub-coded into categories of KT interventions based on the EPOC taxonomy [12].

#### Document review data analysis

All documents were independently coded by two coders. Any discrepancies were reconciled through deliberation until consensus was reached. The research team used descriptive statistics (i.e., counts and proportions) to analyze categorical data and conducted subgroup comparisons for descriptive analyses between regions, as appropriate.

### Phase 2: Survey

#### Survey development

The development of our survey was guided by the Checklist for reporting Results of Internet e-Surveys (CHERRIES) [17]. Survey questions were developed in an iterative manner whereby the project team (i.e., KT Program research team and Project Working Group members from the OSN, HQO, RSNs and LHINs) ensured that the items were aligned with the project objectives. Questions were piloted with 5 stakeholders representing OSN, HQO, and RSN to verify face validity, content sensibility (i.e., comprehensiveness and clarity), survey flow, and timing. After the survey was developed (see Additional file 1), it was translated into French and distributed online in English and French using FluidSurveys™.

#### Participants and recruitment

A purposive sampling strategy was used to identify a wide range of key stakeholders (i.e., LHIN members, stroke program managers, medical directors, hospital chief executive officers (CEOs), hospital chief financial officers (CFOs), organizational administration leads, other senior leaders and teams within hospitals, RSN members, and frontline clinicians and staff (i.e., nurses, physiotherapists, occupational therapists, pharmacists, and speech-language pathologists)) across Ontario. These stakeholders were identified based on input from the Project Working Group. Initial email invitations were sent to stakeholders from someone within their circle of contact (e.g., members of our working group, regional program managers, and senior leaders) along with a link to the online survey. To facilitate recruitment, a briefing note about the evaluation project and survey was distributed to stakeholders via email and during monthly stakeholder meetings. To optimize the survey response rate and the representation of different stakeholder groups, Dillman’s reminder strategy was used to send 3 follow-up email reminders to stakeholders at 1-, 3- and 7 week intervals after the initial survey launch [18]. Data were collected between May 12, 2015 and July 31, 2015.

#### Survey data analysis

Analyses of quantitative survey data (e.g., Likert scale questions) were performed using SPSS version 22.0 to calculate descriptive statistics for all survey items (i.e., proportions for categorical items, means with standard deviations for continuous items, and medians with interquartile ranges for ordinal items). In order to consider the largest possible sample size for the analyses, we included all available data from complete and incomplete surveys. For this reason, the denominators vary by survey item and gradually declined toward the end of the survey. A chi-square test was used to compare the differences between participants who completed the survey and those who did not. Results demonstrated that there were no differences between participants who completed or did not complete the survey by perceived organizational priority or awareness of the handbook. French responses were translated and then merged with the English survey data for analysis. All text responses to open-ended survey items were exported from FluidSurveys™ for qualitative data analysis. This was performed manually whereby responses were grouped into categories using a content analysis approach [19]. Analysis was performed by one member of the study team (KD) and the final results reviewed for validity by a second member (CM).

### Phase 3: Interviews and focus groups

#### Interview and focus group guide

The TDF and CFIR were used to develop framework-informed interview and focus group questions aimed at eliciting data on behaviours, perceptions, and implementation context for stroke QBPs. Findings from the survey data (e.g., broader themes that emerged) also helped to inform the development of the interview guide (see Additional file 2). The guide was adapted for five stakeholder groups (i.e., LHIN, decision support team, clinical team, CEO/CFO, and administrative staff (i.e., administrative director, vice-president, chief of staff, and senior leadership)). An iterative approach to data collection was employed, whereby data from completed interviews were used to revise the guide to ensure it met study objectives.

#### Participants and recruitment

A sequential purposive quota sampling approach was employed whereby only participants from Phase 2 who indicated a desire to participate in Phase 3 were invited to participate [20, 21]. In consultation with the Project Working Group, an anonymized list of eligible Phase 3 participants (identified only by their professional roles) was reviewed and participants were purposively selected to cover a range of roles and regions across the province (e.g. LHIN region, hospital and hospital characteristics (QBP hospital size, stroke distinction, and presence of a stroke unit)). For convenience, regional program directors and district stroke coordinators (RSN staff) were given the option of participating in focus group sessions prior to their monthly Advisory Meetings. Interviews and focus group sessions were conducted between September 2015 and February 2016.

#### Interview data collection and analysis

Telephone interviews and in-person focus groups were conducted by experienced facilitators (CM, KD, RS). Facilitators were knowledgeable in the types of KT activities and interventions used to support QBP implementation which helped to ensure a true account of the participant’s experience was obtained. Interviews and focus groups were 60 and 90 min in length, respectively, and were audio recorded and transcribed verbatim. We used a framework approach to analyze the data while focusing on specific areas of interest. Key steps to framework analysis include: familiarization of the data, identification of a thematic framework, indexing, charting, and mapping and interpretation [22]. Two qualitative analysts (CM, RS) independently reviewed the transcripts to develop an initial coding framework, which was piloted on a small number of transcripts. The framework was then further refined and modified and applied to the remaining transcripts using a modified audit, consensus coding approach [23]. Initially transcripts were divided into groups and coded by two analysts in sequential rounds using NVivo 10 [24]. At the end of each round, inter-rater reliability (IRR) between analysts was calculated using the Kappa coefficient and any discrepancies were discussed and resolved during consensus meetings. Once the number of discrepancies decreased (i.e., Kappa coefficients ≥0.6) the remaining transcripts were coded in further rounds by one analyst, and coding verified on one randomly selected transcript per round by the secondary analyst. IRR was calculated for the audit transcript and if any discrepancies arose, the analysts discussed and resolved themes until the Kappa coefficient was ≥0.6. Reporting of the data was guided by the consolidated criteria for reporting qualitative research (COREQ) [25].

### Triangulation

The document review and surveys provide complementary information about the types of KT interventions used at a regional and organizational level. The document review data presented work plans specifically for each *region*, but did not provide data at an organizational level. The surveys were completed by members of the RSN, LHIN, clinical team, hospital leadership, decision support, and clinical team. Therefore, the surveys primarily captured *organizational*-level data, but the information were aggregated by stroke region to compare the OSN work plans to see whether the organizational activities reflected regional work plans. Interviews and focus groups provided individuals’ views on the planning for and implementation of stroke QBPs and were used to explore implementation at the organization level more deeply. A multi-source (i.e., document review, surveys, interviews, and focus groups) mixed-method triangulation technique was applied to analyze the data. We used concurrent data triangulation to seek convergence, inconsistency, or contradiction between two data sources, surveys and OSN work plans [26–28]. All data collected were combined and analyzed using a meta-matrix [29]. The use of a meta-matrix facilitated a strong visual anchor for pattern recognition and for confirming, elaborating, and identifying contradicting/discrepant views between data types within a single case and across all cases.

### Ethics and consent

Ethical approval was obtained from St. Michael’s Hospital Research Ethics Board (REB #15-.

062). Informed consent was obtained from all participants prior to the survey and interviews/focus groups.

## Results

### Phase 1: Document review

Eight work plans were received (i.e., seven work plans from seven RSNs and one combined work plan from three RSNs), representing 10 of the 11 regional networks. Through data abstraction, we identified a total of 446 deliverables. Each deliverable was mapped to one of the 12 KT activities (see Table 2). The number of deliverables in each work plan varied by region from 12 to 175, with a mean of 55.8 (standard deviation [SD] = 58.6). The most common deliverables were categorized as ‘other – dissemination’ (24.2%; *n* = 108), ‘select, tailor, implement interventions – *implementation*’ (22.6%; *n* = 101), ‘select, tailor, implement interventions – *implementation planning*’ (15.0%; *n* = 67), or ‘knowledge tools’ (10.5%; *n* = 47). Few deliverables were identified for each of the following KT activities: ‘assessing barriers/facilitators to knowledge use’ (0%; *n* = 0), ‘adapting knowledge to local context’ (0.1%; *n* = 4), ‘sustain knowledge use’ (2.7%; *n* = 12), or ‘evaluate outcomes’ (3.1%; *n* = 14).

**Table 2:** KT activities by region from work plans

KT activities	OSN region								# KT activities	% of total (%)
	A	B	C	D	E	F	G	H		
Knowledge tools	3	1	1	1	2	21	17	1	47	10.5%
Identify problem/ identify, review, select knowledge	0	0	1	0	1	14	3	1	20	4.5%
Adapt knowledge to local context	1	0	0	0	1	1	1	0	4	0.9%
Assess barriers/facilitators to knowledge use	0	0	0	0	0	0	0	0	0	0.0%
Select, tailor, implement interventions - implementation planning	6	3	1	3	10	12	29	3	67	15.0%
Select, tailor, implement interventions - implementation	8	2	6	5	5	28	30	17	101	22.6%
Monitor knowledge use	0	1	5	1	5	6	16	4	38	8.5%
Evaluate outcomes	3	0	0	0	1	3	7	0	14	3.1%
Sustain knowledge use	2	3	0	0	0	0	7	0	12	2.7%
Other – dissemination	16	3	0	1	2	23	55	8	108	24.2%
Other – stakeholder engagement	4	2	0	1	4	7	10	7	35	7.8%
TOTAL # deliverables per region	43	15	14	12	31	115	175	41	446	100.0%

e.g., *To preserve anonymity, each region has been assigned a letter*

We categorized each deliverable from the ‘select, tailor, implement interventions – *implementation*’ activity using the EPOC taxonomy for KT interventions (see Table 3).

**Table 3:** KT interventions

KT interventions	Phase 1: OSN work plan deliverables (n = 101)^+^	Survey data from frontline clinician and senior leadership (n = 357)*	Survey data from CEO/CFO, DS, RSN (n = 109)*
Organizational KT interventions			
Accreditation	3	42	24
Changes to affiliation status of hospitals and other facilities	0	NRO	15
Changes in physical structure, facilities, and equipment	0	NRO	17
Changes in quality monitoring system	0	NRO	40
Changes in setting/site of delivery	9	NRO	37
Skill mix changes	4	62	31
Professional KT interventions			
Audit and feedback	2	118	NRO
Champion/opinion leader	5	148	NRO
Continuity of stroke care	12	88	NRO
Educational meetings	25	154	NRO
Local consensus processes	0	109	NRO
Multidisciplinary teams	3	113	NRO
Patient educational materials	5	136	NRO
Reminders	0	61	NRO
Revision of professional roles	1	87	NRO
Staff educational materials	7	145	NRO

*NRO = not a response option;* ^*+*^ Does not equal number of deliverables because five deliverables were mapped to two KT interventions; *Response options were not mutually exclusive, respondents could select multiple KT interventions

Of the 101 deliverables, 30 could not be categorized because of insufficient information. For example, the deliverable “assist with the implementation of in-hospital code stroke processes at hospitals in the region” was too broad to categorize. The remaining 71 deliverables were categorized into 11 EPOC KT interventions: educational meetings (*n =* 25), continuity of care (*n =* 12), changes in setting/site of delivery (*n =* 9), staff educational materials (*n =* 7), champion/opinion leader (*n =* 5), patient education (*n =* 5), skill mix changes (*n =* 4), accreditation (*n =* 3), multidisciplinary teams (*n =* 3), audit and feedback (*n =* 2), and revision of professional roles (*n =* 1). Five deliverables were categorized as multiple KT interventions, for example: “continue to facilitate and support the implementation of standardized resources at a regional level to enable stroke flow throughout the continuum of care” was considered both an ‘educational materials’ and a ‘continuity of care’ strategy. Spreading general information about QBPs was typically categorized as ‘other – dissemination’ KT activities, and therefore were not mapped to the EPOC KT interventions, unless the description referenced stroke QBP implementation specifically; for example, “support the District Stroke Centres and Regional Stroke Centres in implementing the screening tools, including monitoring and addressing implementation challenges as they arise”.

### Phase 2 and 3: Surveys, interviews, and focus groups

#### Participant demographics

Four hundred and eighty-nine staff members responded to the survey and were included in the analysis. Due to the sampling method, we were not able to collect an accurate response rate. We had representation from every LHIN, but not from every hospital within each LHIN. Among the respondents, 66 (13.5%) were from the senior leadership team, 22 (4.5%) were CEOs/CFOs, 38 (7.8%) were from the decision support team, 291 (59.5%) were from the clinical team, 49 (10.0%) were from the RSNs, and 23 (4.7%) were from LHINs. For this analysis focused at a regional/hospital level, responses at the LHIN level were removed (*n* = 23). As participants received different questions based on their role, the number of responses by question varied. There were a total of 44 interview/focus group participants; six LHIN representatives, one CFO, nine senior leadership members, two decision support members, 17 clinical team members, and nine RSN members.

#### KT interventions

Based on their roles, respondents were asked about the types of KT interventions they were aware of in their organization (see Table 3). The most common KT interventions reported by frontline staff and senior leadership team (*n* = 357) included: educational meetings (43.1%; *n* = 154), presence of a leader to champion improvements (41.5%; *n* = 148), and distribution of staff educational materials (40.6%; *n* = 145). Similar findings emerged from the interviews, for example a member from the senior leadership team cited:

*“An interesting fact came up with some solutions of having some champions on the floor available to continue meeting this QBP target for our patients, and we’ve actually improved getting to that on the quality, on the day needed, within the first twenty-four hours about seventy per cent of the time, which was a lot different than hitting and missing it about thirty per cent of the time.” – Senior leadership 015.*

At an organizational level, respondents (*n* = 109) reported making changes to the setting/site of service delivery (e.g., moving a service from one location to another) (33.9%; *n* = 37); changes to the organization’s quality monitoring system (e.g., how decision support data is used for quality improvement) (36.7%; *n* = 40); changes to staff organization (e.g., relocation/restructuring of staff within organization) (28.4%; *n* = 31); changes in the physical structure, facilities, and equipment (15.6%; *n* = 17); Accreditation Canada’s Stroke Distinction program (22.0%; *n =* 24); and changes to affiliation status of hospitals and other facilities (13.8%; *n* = 15).

#### Resources developed for stroke QBP implementation and dissemination

As part of stroke QBP implementation and to support the dissemination of information about stroke care in the province, hospitals were provided access to three reports, the Ontario Stroke Report Card, the Ontario Stroke Evaluation Report, and the QBP baseline report. Respondents were asked whether or not they had reviewed each of the reports. Less than half of respondents for this question (*n* = 467) reported reviewing the 2012/2013 Ontario Stroke Report Card (43.5%; *n =* 203) and the 2014 Ontario Stroke Evaluation Report (37.9%; *n =* 177). Approximately 17.3% (*n =* 81) had reviewed the QBP baseline report, and 21.4% (*n* = 100) had not reviewed any of the reports. During interviews, participants provided further details regarding the report, such as:

*“We created a scorecard, which is kind of like a report card, and we report every month, and we actually send that to the team. So, the team is able to monitor their own performance, and… that’s something we recently implemented a few months ago, and it’s still very fresh, and very new, but we’re trying to use that as a way to kind of measure, you know, what we’re doing on a monthly basis, and it helps us guide where our priority work is.” – Senior leadership 039.*

Additionally, hospitals had access to several other resources to support stroke QBP implementation, for example clinical pathways and checklists. The majority of respondents for this question (*n* = 444) reported using at least one resource (63.1%; *n* = 280), specifically: order sets (56.1%; *n =* 249), clinical pathways (52.9%; *n =* 235), protocols (38.1%; *n =* 169), process improvement approaches (32.2%; *n =* 143), medical directives (16.7%; *n =* 74), QBP checklists (15.8%; *n =* 70), and management tools (15.8%; *n =* 70). Moreover, participants spoke of the importance of these resources during interviews and focus group sessions.

*“We viewed, at the LHIN level, various order sets, because of the different types of strokes: ischemia, hemorrhagic, and also the TIA, and the work that was being developed there was also brought in internally here in the organization, in order for us to modify our order sets to align with the stroke pathway. So, that’s what we did in order to get the unit ready in implementing the QBP, as well as the organization.” - Clinical Team 022.*

#### Perceived level of success with stroke QBP implementation

Respondents perceived their organizations to be moderately successful (providing ratings from 4 to 6 on a scale from 1 to 7) in implementing the stroke QBPs by their responses to the survey question (median = 5; interquartile range = 4–6; *n* = 335, Likert 1 to 7) and feedback received during the interviews.

*“I think we’ve been a hundred per cent successful on the parts that we’ve implemented. I think there’s still, you know, a lot of work to do to maintain 1) some parts that we haven’t, and 2) to maintain and sustainability of the stroke QBP, and keeping it to the forefront, but I envision after the end of this, if you do this for sustainability for the next five, or ten years it becomes inherent practice. We no longer call it a stroke QBP handbook. It’s just best practice.”- Senior leadership 015.*

#### Perceived determinants influencing stroke QBP implementation

##### Facilitators

Respondents reported three CFIR constructs and sub-constructs at the organizational level that were perceived to have influenced the effectiveness of the implementation. Specifically, respondents expressed that key facilitators in advancing the uptake of stroke QBPs were: ‘the implementation climate – relative priority’, ‘readiness for implementation – leadership engagement & available resources’ and ‘cosmopolitanism (i.e., networks with external organizations)’. For example, respondents reported that stroke QBPs were seen as a priority in their organization (median = 6; interquartile range = 5–7; *n* = 458, Likert 1 to 7). Respondents also indicated the importance of engaging leaders and managers early on in the implementation and that this early commitment was beneficial in shaping the implementation plan, and ensuring accountability within the organization. Furthermore, respondents said that it was valuable to have networks with well-established organizations such as the OSN and RSNs to leverage their existing structures and resources.

*“We [the RSN] play a big support role…specifically, supporting business cases to drive forward quality-based procedures. For example, for integrated stroke units, and that’s not only within our own organization, but supporting our partner community organizations, providing them with information, with statistics, with all of those types of things, and we also drive collaboration across the continuum.” – RSN member 001.*

Additionally, several respondents expressed that the LHIN’s involvement was key in information-sharing and fostering collaborations between stakeholders and other institutions. Respondents identified CFIR constructs directed to the individual level such as the ‘knowledge and beliefs about the intervention’, ‘self-efficacy’, and the TDF construct ‘skills’ as facilitators to implementation. Respondents reported that they that were aware of the rationale for stroke QBPs (median = 6; interquartile range = 6–7; *n* = 252)*,* they recognized the benefits of stroke QBPs (median = 6; interquartile range = 6–7; *n* = 248)*,* and that they had the skills to implement stroke QBPs (median = 6; interquartile range = 5–6; *n* = 249).

##### Barriers

Perceived barriers to implementation at an organizational level included the CFIR construct and sub-construct ‘readiness for implementation - available resources’ and the TDF domain ‘environmental context and resources’. Specifically, most respondents (62.9%; *n* = 110) reported a lack of funding to fully implement stroke QBPs and 56.0% (*n* = 98) of respondents cited resource implications for supporting multiple QBPs as a barrier. Furthermore, respondents perceived that they had not been adequately trained on how to carry out the recommended stroke QBPs (median = 4; interquartile range = 3–5; *n* = 239). The key barrier to implementation at an individual level was the CFIR construct ‘knowledge and beliefs about the intervention’. Several interview respondents described their concerns about the funding formula associated with stroke QBPs, specifically, concerns over organizations that were not included as part of the HSFR model (i.e., where costs could not be extricated) and where funding was based on volumes, and not based on quality outcomes.

*“I think for some hospitals they don’t necessarily have the resources to implement QBPs – and we certainly don’t either but I think that’s becoming difficult as you’re getting more and more of them [QBPs]. How do you find the resources to start to implement all this change simultaneously?” – Senior leadership 013.*

### Triangulation

Triangulation of work plans and survey data indicated a mix of consistencies and inconsistencies in what was reported at the regional, organizational, and frontline level. At an organizational level, there were a similar number of frontline staff and leaders reporting the use of accreditation and skill mix changes at their hospitals. However, most regional work plans did not report on the use of accreditation and skill mix changes. A large proportion of the leaders reported in the survey that they made changes to their organization’s setting/site of delivery while this was underreported in the work plans. Major discrepancies occurred between frontline staff and RSN work plans where frontline staff reported frequent use of audit and feedback, champions, local consensus processes, and multidisciplinary teams, while these were infrequently mentioned in work plans.

There were some consistencies across work plans and survey responses from frontline staff; specifically, both frequently mentioned the use of KT interventions such as continuity of care and educational meetings. Additionally, both work plans and frontline staff rarely reported the use of reminders as KT interventions. The difference between reported use of patient and staff education materials between work plans and surveys is likely because most educational materials in the work plans were coded as “dissemination” activities in the KTA, so not mapped to the EPOC taxonomy.

At a regional level, items identified in the work plan were compared to survey responses from front line staff across the eight RSN regions (see Table 4). If a KT intervention was mentioned once in a region’s work plan, it was checked as ‘included’ in that region. If at least 50% of frontline staff in that region indicated “yes” for a KT intervention, it was selected as ‘implemented’ in that region. There were a total of 12 types of KT interventions to compare across eight regions. The number of KT interventions that were identified in work plans varied from one to nine interventions depending on the region, indicating variation *between* regions. Of the KT interventions that were identified as being ‘implemented’ from surveys, there were five out of a possible 37 instances that they overlapped with KT interventions from three regions’ work plans. The irregular overlaps indicate variability between frontline clinicians *within* regions. In instances when KT interventions did align, they included KT interventions such as ‘changes in setting/site of delivery, ‘educational materials’ and ‘educational meetings’.

**Table 4:** KT interventions by regions

	A		B		C		D		E		F		G		H		Total
Source	W	S	W	S	W	S	W	S	W	S	W	S	W	S	W	S	
Accreditation		✓			✓								✓		✓		4
Audit and feedback													✓				1
Champion/opinion leader		✓									✓		✓				3
Changes in quality monitoring system		✓															1
Changes in setting/site of delivery	✓		✓				✓		✓	✓	✓	✓	✓				8
Continuity of care	✓				✓		✓				✓		✓		✓		6
Educational materials											✓		✓	✓	✓		4
Educational meetings	✓				✓				✓		✓	✓	✓	✓	✓		8
Multidisciplinary teams													✓				1
Patient education					✓						✓		✓		✓		4
Revision of professional roles							✓										1
Skill mix changes							✓		✓		✓				✓		4

e.g.*, To preserve anonymity, each region has been assigned a letter; ✓ indicates that the regional workplan (W) included the KT intervention or if the majority (> 50%) of survey respondents from that region (S) indicated they used the KT intervention*

## Discussion

Overall, stroke QBP implementation in Ontario was perceived to be moderately successful (ratings of 4–6 on a scale from 1 to 7). A variety of KT interventions were used at the *individual* level with frontline clinicians to align their practice with QBP recommendations, at the *organizational* level with senior leaders and QBP implementers, and at a *regional* level through the LHINs, OSN, and RSNs. Through this evaluation, a common theme has been the large variability between health care organizations and RSNs. For example, some organizations focused almost exclusively on organizational-level changes, others on individual-level changes, and others on both. Noted barriers to implementation included lack of organizational readiness, and contextual issues such as lack of funding and training, limited knowledge of QBPs, and not believing in their value. Key facilitators were the relative priority of stroke QBPs, leadership engagement, and connections with other organizations and the OSN/RSNs.

Our findings are similar to other published studies on the implementation of stroke best practice guidelines and evidence-based care [9, 30] in related settings. These studies described practice changes implemented at either the *organizational* or *individual* level. While findings from these studies showed improvements in patient outcomes, the studies reported that implementing major practice changes in health care organizations is complex and changes need to occur at all levels across the system [31].

At a regional level, RSNs conducted a range of activities to support QBP implementation, providing hospitals with resources, leadership, and guidance throughout implementation. When KT activities were mapped onto the KTA, we were able to identify ‘what was implemented’. The most common activities reported in work plans were ‘using dissemination strategies’, ‘KT interventions’, and ‘creating tools’ (e.g., sharing educational tools with clinicians across hospitals) indicating where efforts were focused. As such, most regions did not emphasize implementation but rather focused on dissemination. We were unable to categorize approximately 30% of the KT interventions due to insufficient detail. This finding underscores the importance of specifying KT interventions both from an operationalization perspective and from a research perspective to enable measurement and replicability. KT interventions targeted to clinicians were the primary approach used to implement stroke QBPs. There was a general underreporting of KT interventions in work plans, indicating that work plans may have been further tailored by each organization and clinical leader. However, it may also indicate a lack of clarity or fit of the original work plans to the QBP implementation process, which perhaps could have been prevented by providing organizations with additional operationalized details on *how* to implement QBPs.

When comparing regional work plans to survey responses within a region, there were large discrepancies between the regions and between the RSN team and frontline clinicians. Not surprisingly, no work plans were the same for any region, and the number of different types of KT interventions reported varied from one to nine, which could have impacted on how effectively and efficiently implementation was achieved. Additionally, of the 37 KT interventions identified in work plans, five were reported by both RSNs and clinicians. Though variations could mean that a flexible and tailored approach was considered in implementing stroke QBPs, it is not clear what impact the tailoring had on implementation and outcomes.

A key stage in the KTA cycle that was underrepresented in regional work plans is assessing barriers and facilitators to change. Behaviour change is a complex process, further complicated when multiple people, organizations, and systems need to change. In order to effectively support behaviour change, it is imperative to understand *why* people are/are not changing. Conducting a barriers and facilitators assessment is one way to accomplish this task, which can serve as the basis for selecting KT interventions that support behaviour change [31]. Alternatively, barriers and facilitators can be identified from existing studies [8–10] and prioritized for the local context; this process saves time and would allow the regions to focus on linking barriers and facilitators to behaviour change theory to understand the mechanism of change that is likely to result in practice change. Once the barriers and facilitators are linked to behaviour change theory, appropriate dissemination and KT interventions can be selected that are based on high-quality evidence and address the underlying mechanisms of change [31]. For instance, several barriers and facilitators to stroke QBP implementation were identified in both the survey and interviews/focus group sessions. Key barriers were lack of resources (e.g., funding and training) to properly implement stroke QBPs, simultaneous implementation of multiple QBPs, and knowledge and beliefs about the intervention. Key facilitators were the presence of supportive networks, the perception that stroke QBP implementation was a priority among staff, the availability of certain resources, and leadership engagement. Leadership engagement may have been enhanced by linking QBP implementation to funding. Funding and leadership engagement are key facilitators to implementation [32, 33].

A second gap from the KTA framework noted in the work plans was evidence of planning for sustainability. Sustainability is the continued delivery of KT interventions and the maintenance of stroke best practices and outcomes [34]. Since planning for sustainability is related to actual sustainability [35], there is an opportunity to explicitly plan for the sustainability of the KT strategies and the QBP changes. Unfortunately there is very little guidance available on how to sustain the delivery of KT interventions, behavior change, and outcomes [36]. There are a number of measures and tools available and emerging research to show that the use of these that could support local sustainability efforts [37–39].

To support others implementing stroke guidelines, four recommendations were provided to increase the effectiveness of QBP implementation strategies: 1) assess barriers and facilitators to clinician, organization, and system changes; 2) use theory and evidence to drive implementation and dissemination strategy selection; 3) maximize on economies of scale in tool development, develop centralized tools that can be adapted regionally and share regionally developed tools and allow for adaptation; and 4) plan for sustainability (see Additional file 3 for the rationale and considerations for each recommendation).

This study has several limitations. First, we were unable to determine survey response rate due to the sampling strategy. However, participants who responded represent the different LHINs and stakeholder groups. Second, we do not have data on outcomes from the QBP implementation. Outcome data analysis is underway; in the future these results will be used in conjunction with those outcomes. Third, the interviews and survey responses may not be generalizable to all health care providers and leaders in Ontario. However, this was a relatively large sample size for both the survey and interviews/focus groups and the participant demographics are representative of the target population.

## Conclusion

Frontline clinicians, senior leaders, and RSNs are all working to implement QBPs across the province of Ontario. We identified the types of activities and KT interventions used to support stroke QBP implementation and the key determinants that influenced their uptake. While there is some consistency across levels, there is also a large amount of variability. Additionally, there are gaps in some of the key steps or stages, such as assessing barriers and facilitators and planning for sustainability. These present opportunities to streamline future implementation efforts.

## Additional files

Additional file 1: Phase 2 Master list of survey questions (PDF 265 kb)

Additional file 2: Phase 3 Interview Guide (PDF 465 kb)

Additional file 3: Recommendations for QBP Implementation Strategies (DOCX 30 kb)

## Abbreviations

CADTH: Canadian Agency for drugs and Technologies in Health
CEOs: Chief Executive Officer
CFOs: Chief Financial Officer
EPOC: Effective Practice and Organization of Care
HQO: Health Quality Ontario
HSFR: Health Systems Funding Reform
KT: Knowledge Translation
KTA: Knowledge to action model
LHIN: Local Health Integration Networks
OSN: Ontario Stroke Network
QBPs: Quality Based Procedures
RSNs: Regional Stroke Networks
