# Supplementary material for: Supporting the implementation of stroke quality-based procedures (QBPs): a mixed methods evaluation to identify knowledge translation activities, knowledge translation interventions, and determinants of implementation across Ontario
Source: BMC Health Serv Res. 2018 Jun 18;18:466. doi: 10.1186/s12913-018-3220-9 (PMC6006745; doi:10.1186/s12913-018-3220-9)
Supplement: Supplementary file 1 — Phase 2 Master list of survey questions (PDF 265 kb) [file 12913_2018_3220_MOESM1_ESM.pdf]

Additional file 1: Phase 2 master list of survey questions

**Survey Questions**
1. Please select the category that best describes your current primary job position/title.<br><br>○ Hospital Organization CEO<br>○ Hospital Organization CFO<br>○ LHIN CEO<br>○ LHIN Senior Director of Performance<br>○ LHIN Senior Director of Planning<br>○ LHIN Representative on the Regional Stroke Committee<br>○ Regional Program Director<br>○ District Stroke Coordinator<br>○ Regional Team Member<br>○ Vice President of Patient Care<br>○ Chief of Staff<br>○ Administrative Lead of Decision Support<br>○ Data Analyst<br>○ Health Records Data Quality Lead<br>○ Administrative Director<br>○ Program Manager<br>○ Physician Lead<br>○ Allied Health Practice Leader<br>○ Stroke Neurologist/Specialist<br>○ Nurse<br>○ Pharmacist<br>○ Physiotherapist<br>○ Occupational Therapist<br>○ Speech-Language Pathologist<br>○ Psychologist
2. Do you have any other roles you would like us to know about? [Yes – please provide details /
3. Please indicate the area/department that you currently work in (select all that apply)

a. Emergency department<br>b. Acute care<br>b. i) Inpatients<br>b. ii) Outpatients<br>b. iii) Both inpatients and outpatients<br>c. Rehabilitation<br>c. i) Freestanding (rehabilitation facility/unit not attached to acute care)<br>c. ii) Non-freestanding (rehabilitation facility/unit attached to acute care)
4. How long have you been at your current primary job position/title?<br>a. under 2 years<br>b. 2 to 10 years<br>c. 11 to 20 years<br>d. 21+ years
5. Please indicate the region you work in and the hospital that you most frequently work at. If you are not sure of the LHIN region, select "I don't know" and you will see the full list of hospitals to select from.<br>Please click on the box below to select your region. A second box will then appear with a list of hospitals to select from.
6. Which region do you represent?
7. Are you a member of the LHIN Local Partnership Committee? [yes/no]
8. To what extent do you feel implementing stroke QBPs is a priority in your organization?<br>[Not at all – To a great extent]
9. How has your organization communicated stroke QBPs implementation as a priority? (Select all that apply)<br>a. Through its corporate Quality Improvement Plan<br>b. Through its corporate strategic plan and associated implementation plan, improvement priorities, tactics and milestones<br>c. Through program tactics or action plans<br>d. Through newsletters or other hospital communications<br>e. Through town halls or meetings with staff<br>f. Other (please specify) ________________________
10. In preparation for stroke QBPs implementation, has your organization…<br>a. Assessed its current state of stroke care (e.g., healthcare providers, clinical pathways, availability of data)? [Yes/No/I don't know]

b. Conducted a gap analysis (e.g., compared the current clinical process for stroke and the stroke QBPs pathway, identified opportunities for improvements)?<br>c. Assessed the impact of the stroke QBPs funding changes?<br>d. Assessed quality indicators identified in the QBPs Clinical Handbook for Stroke?<br>e. Assessed the operational and clinical changes (e.g., staffing)?<br>f. Established new committees or cross-functional teams?<br>g. Other (please specify) __________________
11. For the implementation of stroke QBPs has your organization developed a plan to: (Select all that apply) [Yes/No/I don't know]<br>a. Address the gaps in practice between the current clinical process for stroke and the stroke QBPs pathway?<br>b. Address the impact of the stroke care QBPs funding changes?<br>c. Utilize the quality indicators to gauge performance?<br>d. Address the operational and clinical changes with respect to stroke care?<br>e. Monitor and measure both the progress and outcomes of stroke QBPs implementation?<br>f. Sustain and maintain the changes as a result of stroke QBPs implementation?<br>g. Other (please specify) _____________________
12. Has your organization identified tools (e.g., clinical pathways, protocols, order sets, etc.) to assist with the organizational structure and set-up for stroke QBP implementation? [Yes/No/I don't know]<br>a. Please select the tools/resources that you are aware of and which have been used to facilitate the implementation of stroke QBPs:<br>a. Clinical pathways<br>b. Protocols<br>c. Order sets<br>d. Medical directives<br>e. Utilization management tools<br>f. Process improvement approaches<br>g. QBP checklists<br>h. Other (please specify)
13. When did your organization start to roll out stroke QBPs?<br>Please include the year (e.g., 2012, 2013, 2014) and season (e.g., spring, summer, fall, winter). If you are unsure/unaware or have not yet begun, please indicate as such.
14. To what extent do you feel that in your organization stroke QBP pathways are now the standard ways of providing stroke care? [Not at all – To a great extent]
15. What has been your involvement with stroke QBP implementation (i.e., role and responsibilities)?

16. Which of the following reports have you reviewed? (Select all that apply)
    - 2014 Ontario Stroke Evaluation Report
    - 2012/2013 Ontario Stroke Report Card
    - QBP baseline report
    - None of the above
    - Other (please specify) ______________________

17. Below is a list of recommended stroke QBP practices in the care setting you indicated you work in (i.e., emergency department, acute inpatient care, and inpatient rehabilitation). Please indicate the stroke care practices that are included in your organization.

[Not planned / In progress / Implemented / I don't know/NA]

Emergency Department
- Regional protocol for medical redirect where indicated and timely arrival by ambulance
- Protocol for provision of acute thrombolytic therapy
- Protocol for timely access to brain imaging (CT or MRI)
- Standardized stroke care pathway
- Standardized admission stroke order set
- Swallowing screening protocol
- Patients with atrial fibrillation are prescribed or recommended anticoagulant therapy on discharge from the ED
- Patients without atrial fibrillation receive carotid imaging prior to discharge from the ED
- TIA - timely vascular imaging of brain and neck arteries
- TIA - referral to Stroke Prevention Clinic

Acute Care
- Standardized stroke care pathway
- Patients should be admitted to a specialized, geographically defined hospital unit dedicated to the management of stroke patients
- Assessment by the interprofessional care team within 48 hours
- Alpha FIM instrument is completed on Day 3 of admission
- Inpatient rehab referral initiated based on standardized rehab triage guidelines and Alpha FIM score
- Early mobilization
- Swallowing screening protocol
- Length of stay: Ischemic strokes 5 days
- Length of stay: Hemorrhagic strokes 7 days
- Depression screening protocol
- Patient/family stroke education is provided in a standardized manner

Teaching and support in secondary prevention of stroke including smoking cessation where indicated
Patients with atrial fibrillation are prescribed or recommended anticoagulant therapy by discharge
Patients without atrial fibrillation receive carotid imaging by discharge

Rehabilitation Unit

Patients should be admitted to a specialized, geographically defined hospital unit dedicated to the management of stroke patients
Can be admitted 7 days/week
Assessment by the interprofessional care team within 48 hours of admission
Stroke patients should receive, through an individualized treatment plan, at least 3 hours of direct task-specific therapy
Stroke patients should receive the above therapy for at least 6 days a week
Patient/family stroke education is provided in a standardized manner
Method developed to prospectively monitor and work to attain RPG Length of Stay targets

18. Are you aware of the stroke QBP Clinical Handbook for Stroke?

Yes, I am aware of it and have read it
Yes, I am aware of it but have not read it
No, I am not aware of it

19. Please indicate the degree of implementation of the QBPs Clinical Handbook for Stroke at your organization.

[Very low – Very high]
I don’t know/Unaware

20. Please indicate which strategies (if any) your organization has used to improve the uptake of stroke QBPs best practices outlined in the QBPs Clinical Handbook for Stroke. (Select all that apply)

a. Distribution of staff educational materials (e.g., distribution of published or printed recommendations for stroke care, clinical practice guidelines, audio-visual materials and electronic publication)
b. Distribution of patient educational materials (e.g., distribution of published or printed recommendations for stroke care, audio-visual materials and electronic publication)
c. Provision of educational meetings/sessions (e.g., small group learning activities, videoconferences, webinars, and workshops)
d. Local consensus processes (e.g., meeting with those who will be impacted by stroke QBPs implementation to ensure that they agreed that the chosen stroke care approach is appropriate)
e. Presence of clinical/administrative leader to champion improvements (e.g., increase buy-in, and remove barriers)
f. Audit and feedback mechanisms(e.g., OSN Stroke Report Cards, baseline reports, facility based reports)
g. Reminders (e.g., patient or encounter specific information, provided verbally, on paper or on a computer screen, which is designed or intended to prompt a health professional to recall information)
h. Interviews, group discussions, or a survey to identify barriers to stroke QBP implementation and subsequent design of an intervention that addresses identified barriers)
i. Revision of professional roles (e.g., shifting of roles among healthcare professionals, expansion of role to include new tasks)

j. Creation of clinical multidisciplinary teams (e.g., create of new team/team members who work together to care for patients)<br>k. Skill mix changes (e.g., changes in numbers, types or qualification of staff)<br>l. Improvements in continuity of stroke care (e.g., arrangements for follow-up, case management)<br>m. Accreditation Canada's stroke distinction program<br>n. Other, please specify... _______________________
21. Please rate the level of support that each of the following has provided for stroke QBP implementation:<br>a. Evidence-based pathways (e.g., development of Clinical Handbooks)<br>b. Balanced Evaluation (e.g., integrated QBP Scorecard, alignment with Quality Improvement Plans)<br>c. Sector engagement and communication (e.g., Clinical Expert Panels, Clinical engagement sessions, HSFR engagement sessions)<br>d. Knowledge Translation (e.g., Improving and Driving Excellence Across Sectors (IDEAS), tools and guidance documents, HSFR Helpline, HSIMI website)<br>e. Transparency (e.g., published practice standards and evidence underlying prices for QBPs, routine communication and consultation in the field)<br>f. Accreditation Canada's stroke distinction program<br>g. Other (please specify)
22. Please identify the barriers related to the implementation of stroke QBPs. (Select all that apply)<br>a. Management of changes in patient volumes<br>b. Funding inadequate for full implementation of Clinical Handbook Best Practices<br>c. Timing of release of Handbooks<br>d. Clarity of direct or indirect costs inclusions<br>e. Clinical engagement<br>f. Resources implications for supporting multiple QBPs<br>g. Transportation<br>h. Internal hospital resources<br>i. Confusion over QBP cohort definitions<br>j. Lack of timely data<br>k. Inability to replicate Ministry QBP performance indicators<br>i. Other (please specify) _______________________
23. Please indicate which strategies (if any) your organization has taken to implement stroke QBPs. (Select all that apply)<br>a. Changes to the setting/site of service delivery<br>b. Changes in physical structure, facilities and equipment<br>c. Changes to the organization's quality monitoring system<br>d. Changes to affiliation status of hospitals and other facilities<br>e. Changes to staff organization

f. Accreditation Canada's stroke distinction program<br>g. Other (please specify)<br>h. I don't know/Unaware
24. Are you aware of any collaborations/engagement activities that took/are taking place with other institutions and/or agencies during the transition to stroke QBP funding model?<br>[Yes – please provide details/No]
25. Please rate your level of satisfaction with your organization's current stroke QBP practices.<br>[Not at all – Extremely]
26. How are you currently monitoring and evaluating QBP compliance? (Select all that apply)<br>a. QBP Scorecards<br>b. Program-based Scorecards<br>c. Monitoring adherence to order sets<br>d. Monitoring changes in costs using case costing data<br>e. Monitoring indicators at Steering Committee level<br>f. Other (please specify):<br>________________________<br>g. I don't know/Unaware
27. Are there any additional resources/tools that could be developed to support stroke QBP implementation in your organization?<br>[Yes – please provide details / No / I don't know/Unaware]
28. Please rate the extent to which you agree with each of the following statements.<br>Please note, while some questions seem similar they are capturing different aspects of stroke QBP implementation. Please read each statement carefully.<br>a. I am aware of the rationale behind stroke QBPs<br>b. I am not aware of stroke QBPs<br>c. I feel that I have the skills to implement stroke QBPs<br>d. I do not know the stroke QBPs recommendations<br>e. For me, implementing stroke QBPs into my clinical practices will be easy to do<br>f. There are not enough resources to implement stroke QBPs<br>g. I see the benefits to implementing stroke QBPs<br>h. I do not agree with the stroke QBPs recommendations<br>i. I feel that stroke QBPs is a good fit with my routine practices<br>j. I believe that stroke QBPs will not lead to improved healthcare process

k. I feel connected to other clinicians in my organization with whom to discuss stroke QBPs
l. I am afraid that implementing stroke QBPs will negatively impact patient outcomes
m. I have been trained on how to carry out the recommended stroke QBPs
n. I do not have enough time to implement stroke QBPs
o. I feel that in the organization I work there is enough time to implement stroke QBPs
p. I am not motivated to implement stroke QBPs
q. I feel that I have support from senior management with respects to implementing stroke QBPs
r. I am not confident in my ability to implement stroke QBPs
s. I feel that I have support from my colleagues/team of professionals with respect to implementing stroke QBPs
t. I have not been adequately trained to implement stroke QBPs
u. Implementing stroke QBPs is not my role
v. Implementing stroke QBPs is not reinforced as a priority in my organization
w. I forget to implement stroke QBPs
x. I do not have support from colleagues to implement stroke QBPs
y. I do not have support from management to implement stroke QBPs

29. How successful do you perceive the implementation of stroke QBPs in your organization thus far?
[Not at all – Extremely]

30. Besides stroke QBPs, are you aware of other QBPs being implemented or that have been implemented in your organization?
[Yes – please specify: / No / I don’t know/Unaware]
a. Has the implementation of other QBPs helped to facilitate the implementation of stroke QBPs?
[Yes / No / I don’t know/Unaware]
b. What have the lessons learned been? Please provide up to three examples.

31. Was there a difference in the support/approach to the implementation of other QBPs compared to stroke?
[Yes / No / I don’t know/Unaware]
a. If yes, please explain in a sentence or two.
