# Supplementary material for: Supporting the implementation of stroke quality-based procedures (QBPs): a mixed methods evaluation to identify knowledge translation activities, knowledge translation interventions, and determinants of implementation across Ontario
Source: BMC Health Serv Res. 2018 Jun 18;18:466. doi: 10.1186/s12913-018-3220-9 (PMC6006745; doi:10.1186/s12913-018-3220-9)
Supplement: Supplementary file 2 — Phase 3 Interview Guide (PDF 465 kb) [file 12913_2018_3220_MOESM2_ESM.pdf]

Additional file 2: Phase 3 interview guide

**Interview Questions**
1. Can you please begin by providing a brief description of your experience with QBP implementation?
2. Are you familiar with or aware of the rationale behind implementing stroke QBP over other clinical QBPs within your organization?
3. In your opinion, how do you feel your organization prioritizes the implementation of stroke QBPs (i.e., is it a priority or non-priority)?
4. What has been your role with respects to stroke QBP implementation?<br><br>a. Probe - What have been your key tasks/responsibilities in the implementation of stroke QBPs?<br>b. Probe – Do you believe you are capable of implementing the stroke QBPs as required by your role?<br>c. Probe – Do you think you have the necessary resources to implement QBPs (i.e. training/skills, educational materials)?
5. Are you familiar with the QBPs Clinical Handbook for Stroke?<br>(*If participant is not familiar with the clinical handbook see below for follow-up question*)<br><br>a. Probe – Is implementing the recommendations part of your role as [*insert job title*]?<br>b. Probe – How capable (i.e., physiologically and/or physically capable) do you feel in implementing the recommendations in your hospital setting?<br>c. Probe – Do you believe you have the necessary skills required to implement these recommendations?<br>d. Probe – How has the clinical handbook impacted you? Your organization?<br>e. Probe – Please describe some of the strategies that were used to improve the uptake of recommendations in the clinical handbook? In your opinion, which strategies were the most success/least successful? (e.g., use of toolkits to support the application of the clinical handbook)<br>f. Probe – What would be helpful for you in terms of implementing these recommendations?<br><br>[*If participant is not familiar with the handbook*]:<br><br>To give you a bit of background, since the implementation of QBPs, a number of resources such as QBP Clinical Handbooks have been developed to support QBP implementation. These handbooks help to inform health care providers on the pathways that should be implemented to ensure the consistent application of care delivery. Having mentioned that you are unfamiliar with the stroke clinical handbook, how have you come across information with respects to approaches to the implementation of stroke QBP and the care pathway recommendations?
6. How would you describe the organization's capacity with respect to advancing stroke QBP implementation (i.e., sufficient resources)?

7. How would you describe the organization's capability with respect to advancing stroke QBP implementation (i.e., skills)?
8. Can you describe some of the changes that were required to align the organization with stroke QBP recommendations?<br>a. Probe – How have these changes impacted you?<br>b. Probe - How have these changes impacted the organization?
9. Are there any additional changes needed? If so, why? Please provide examples.
10. Since the introduction of QBPs over the last several years, there have been several presentations and engagement sessions given by the MOH, HQO, and OSN to support implementing funding reform…<br>a. Do you recall attending any of the presentations or engagement sessions? If so, which ones did you attend?<br>b. What were your impressions of the presentations/engagement sessions?<br>i. Probe – What did you find most useful/less useful? Why?<br>c. How would you describe the overall affect these presentations/engagement sessions have had on you in terms of stroke QBP implementation?<br>i. Probe – Did the presentations/engagement sessions have an impact on you?<br>d. How would you describe the overall affect these presentations/engagement sessions have had on the organization in terms of stroke QBP implementation?<br>i. Probe – Did the presentations/engagement sessions have an impact on your organization?<br>e. Are there other ways you feel the MOH, HQO, or OSN could disseminate information or engage stakeholders?<br>*[If participant does not recall attending any presentations/engagement sessions provided list of examples]*<br>Examples of presentations and engagement sessions include: (1) an update on the HSFR presented in Oct 2012, by the MOH, (2) HSFR Sector

Engagement Sessions organized throughout the months of March-June 2013, and presented by the MOH and/or HQO, and (3) an overview of stroke care and stroke QBP implementation presented in Nov and Dec 2014 by the OSN.
11. Have there been any forms of support (i.e., support from management/organization) to assist with stroke QBP implementation? If so, what kind of supports do you think were most helpful and which ones were not?
12. Do you have any systems/infrastructure in place to monitor and manage stroke QBP implementation and sustainability in your organization?<br>a. Probe – Has health information and data played a role?<br>b. Probe – Is there a feedback mechanism in place?
13. How would you describe the level of success the organization has made in terms of implementing stroke QBPs?<br>a. Probe – Do you feel the organization has accomplished what they set out to achieve? Why? Please provide examples.<br>b. Probe – What are some specific behaviours/processes that contributed to the organization's success/lack of success?
14. What do you think are some of the factors that influenced the implementation of stroke QBPs?<br>a. Probe – Please describe some of the barriers/challenges to implementing stroke QBPs?<br>b. Probe – Please describe some of the barriers/challenges to the sustainability (i.e., continued use) of stroke QBPs, if any?<br>c. Probe – Please describe some of the facilitators to implementing stroke QBPs?
15. From your perspective, have there been any consequences (positive or negative) as a result of implementing stroke QBPs?<br>a. Probe – Please provide examples of outcomes you expected following stroke QBP implementation.<br>b. Probe – Have there been any unintended consequences of stroke QBP implementation?
16. What advice would you give others –<br>a. Implementing stroke QBPs?<br>b. Implementing other QBPs?
17. If the organization wanted to focus on sustaining stroke QBP implementation and outcomes, what would be some of your suggestions?
