# Supplementary material for: Supporting the implementation of stroke quality-based procedures (QBPs): a mixed methods evaluation to identify knowledge translation activities, knowledge translation interventions, and determinants of implementation across Ontario
Source: BMC Health Serv Res. 2018 Jun 18;18:466. doi: 10.1186/s12913-018-3220-9 (PMC6006745; doi:10.1186/s12913-018-3220-9)
Supplement: Supplementary file 3 — Recommendations for QBP Implementation Strategies (DOCX 30 kb) [file 12913_2018_3220_MOESM3_ESM.docx]

| \| Increasing the Effectiveness of QBP Implementation Strategies \| \| --- \| | |
| --- | --- | --- |
| \| Recommendation \| \| --- \| | \| Rationale and Considerations \| \| --- \| |
| \| 1) Assess barriers and facilitators to clinician, organization, and system changes \| \| --- \| | \| - Behaviour change is a complex process, further complicated when multiple people, organizations, and systems need to change. In order to effectively support behaviour change, it is imperative to understand *why* people are/are not changing. - Conducting a barriers and facilitators assessment is one way to accomplish this task and can then serve as the basis for selecting strategies to support behaviour change. - A barriers and facilitators assessment should go beyond the obvious, surface level barriers to address the root causes of change or inaction. For example, commonly cited barriers include: not having enough time and the change is not a priority. By going deeper, we want to understand why this change is not considered a priority? What are the other priorities? When people talk about the changes not being a priority, is it really a motivation issue? Why they are not motivated to change? - A key opportunity would be to capture the barriers and facilitators to change at all levels (individual, organizational, and system). This information could be used to select appropriate implementation and dissemination strategies. - The current evaluation could serve as the basis of a provincial barriers and facilitator assessment as participants from across Ontario provided key examples of barriers and facilitators encountered when implementing QBPs. - Barriers and facilitators can be mapped to a behaviour change framework, specifically the Theoretical Domains Framework (TDF) (Cane, O’Conner, & Michie, 2012) \| \| --- \| |
| \| 2) Use theory and evidence to drive implementation and dissemination strategy selection \| \| --- \| | \| - Once barriers and facilitators to behaviour change are well understood, they can be linked to behaviour change theory to understand the mechanism of change that is likely to result in practice changes at all levels. - Behaviour change theories are used to predict and understand causal mechanisms; they explain how a person will change (Michie, Atkins & West 2014). - Once the barriers and facilitators are linked to behaviour change theory, appropriate dissemination and implementation strategies can be selected that are based in high-quality evidence and address the underlying mechanisms of change. - This process allows local tailoring of dissemination and implementation strategies, it can also be used to get buy in at the local level. In addition, use of theory to drive the selection of dissemination and implementation strategies would facilitate future evaluations (Nilsen, 2015). - The COM-B (Capability, Opportunity, Motivation – Behaviour) is a behaviour change theory that has been commonly used in guideline implementation, since it focuses on planned behaviour change and has been mapped to the TDF using an expert panel (Michie et al., 2011). - Many interventions focus on capability (e.g., whether people have the knowledge and skills to do something), when motivation is really one of the primary barriers to change. For example, “reminders” are an effective implementation strategy, but are only likely to work if memory is one of the key barriers or facilitators to change, if clinicians are worried about the consequences of changing, they are unlikely to change as a result of a reminder. \| \| --- \| |
| \| 3) Maximize economies of scale in tool development, develop centralized tools that can be adapted regionally/share regionally developed tools and allow for adaptation \| \| --- \| | \| - Evaluation findings have revealed that many RSNs and healthcare organizations are working on very similar tasks, essentially duplicating work. For example, almost all of the regional work plans described an activity that involved developing a protocol or model. - If every region is spending time and resources on developing these protocols and models separately, there are many staff hours that are being allocated to this activity. - An alternative approach may be to develop more centralized tools that can be shared with and adapted by the regions. Tools already developed by the regions could be shared centrally with all regions and RSN teams could be encouraged to adapt existing tools, rather than creating new ones, when possible. - Using an integrated KT approach (Graham et al., 2014), the OSN/RSNs could select a standardized, but tailored approach to supporting stroke QBP implementation. - Tools could be developed based on the identified barriers and facilitators, so that RSNs and hospitals could identify and prioritize their own barriers and facilitators and select implementation strategies to specifically address challenges and opportunities in their local context. Implementation and dissemination resources could then be shared, to reduce duplication. \| \| --- \| |
| \| 4) Plan for sustainability \| \| --- \| | \| - Sustainability, or the continued implementation and maintenance of outcomes is imperative to have a lasting impact (Scheirer et al., 2011). - Since planning for sustainability is related to actual sustainability [Rhoades], there is an opportunity to explicitly focus on sustainability while adopting a consistent process model. - Developing a sustainability plan includes: defining what will be sustained; how much will be sustained; who is responsible for sustaining it; and defining when sustainability starts and ends. There are also tools available, like the National Health Service’s sustainability model that could support local sustainability efforts (Sustainability Model and Guide. (2006-2013)). \| \| --- \| |
